# *Escherichia coli* Survival on Strawberries and Unpacked Romaine Lettuce Washed Using Contaminated Water

**DOI:** 10.3390/foods10061390

**Published:** 2021-06-16

**Authors:** Manreet Bhullar, Bridget Perry, Ana Monge, Lillian Nabwiire, Angela Shaw

**Affiliations:** 1 Department of Horticulture and Natural Resources, Food Science Institute, Kansas State University, Olathe, KS 66061, USA; msbhullar@ksu.edu; 2 Department of Food Science and Human Nutrition, Iowa State University, Ames, IA 50011, USA; perry@iastate.edu (B.P.); analorenamongebrenes@gmail.com (A.M.); nabwiire@iastate.edu (L.N.)

**Keywords:** strawberry, contaminated water, lettuce, uptake, shelf-life

## Abstract

A number of foodborne outbreaks have occurred in the past decade, with higher incidences associated with romaine lettuce and strawberries. Contaminated agricultural water has been reported as the source of microbial contamination in most of these outbreaks. Maintaining the adequate and sanitary quality (0 *E. coli*/100 mL) of agricultural water can be challenging during post-harvest operations such as washing. The study focused on the attachment of generic *E. coli* (Rifampicin resistant) onto romaine lettuce and strawberries, mimicking the produce wash step. The produce was washed with contaminated water, air-dried, and stored in display units for 7 days. The produce was sampled randomly each day and analyzed for the surviving *E. coli* count. The results indicated that *E. coli* can survive in both lettuce and strawberries over extended periods. A survival population of 2.3 log CFU/cm^2^ (day 8) was observed on lettuce with an initial population of 2.8 log CFU/cm^2^ (day 0). On strawberries, the population reduced from 3.0 (day 0) to 1.7 log CFU/cm^2^ (day 7), with an initial *E. coli* concentration of approx. 6 log CFU/mL in the wash water. Strawberry leaves had a higher attachment of *E. coli* than the fruit (*p* < 0.05). In conclusion, romaine lettuce and strawberries washed with contaminated water can cause an outbreak affecting consumers and public health.

## 1. Introduction

Fresh produce (fruits, berries, vegetables, herbs, and tree nuts) provide essential nutrients that are a part of the human diet, such as vitamins, minerals, and many dietary fibers [1,2]. This produce is also increasingly recognized as a source of foodborne outbreaks in many parts of the world [3]. Produce, such as berries and leafy greens, are typically eaten raw and thus do not undergo processing (a kill-step) that would reduce or eliminate microorganisms of public health significance [4]. 

The World Health Organization (WHO) has classified leafy green vegetables, including romaine lettuce, as a priority focus area relating to the safety of fresh produce due to an increase in foodborne outbreaks [5,6]. Three examples of such outbreaks occurred in 2018 and 2019. The first outbreak was with *Escherichia coli (E. coli)* O157:H7 and was linked to romaine lettuce, resulting in 36 states reporting 210 cases, 96 hospitalizations, and 5 deaths [6]. The second outbreak in December 2018 affected 15 states with 59 cases and 23 hospitalizations [7]. In 2019, romaine lettuce from the Salinas Valley growing region in California was found to be contaminated with *E. coli*, sickening 167, with 85 hospitalizations [8]. All of these outbreaks have been traced back to potential contamination from agricultural water. 

Strawberries are another produce item that are typically eaten raw and are widely grown in every state in the U.S. and almost every country in the world [9]. There have been multiple foodborne outbreaks that have been linked to strawberries. Specific examples include outbreaks in 2011 [10] and 2016 [11], with reported cases of *E. coli* O157:H7 and Hepatitis A outbreaks and others linked to *Salmonella* and Norovirus [12,13]. 

The Food Safety Modernization Act (FSMA) Produce Safety Rule (PSR) establishes the regulatory standards for growing, harvesting, storing, and packaging fresh produce. This regulation aimed to establish industry standards that would prevent foodborne outbreaks with produce that is typically eaten raw. Water has been implicated to be a major risk factor in past food outbreaks, and significant attention has been given to the use of quality water on fresh produce farms [14]. The FSMA PSR defines the microbial standards for the quality of the agriculture water that is used for production and post-harvest processing. The FSMA PSR states that irrigation water used for agricultural production cannot have more than 126 cells of generic *E. coli* per 100 mL for safe use on covered crops and zero cells per 100 mL for processing (washing and ice used for storage) on covered produce (such as berries and leafy greens) [15]. The agricultural water requirements by the FDA’s FSMA PSR are under modification, and the new guidelines are expected to be out sometime in 2022.

During post-harvest handling, produce is often washed through immersion into tanks of water that are recirculated. Recirculation is the most common method of water use because of the cost of water and the availability of water [16]. This process allows for the possibility of contaminated produce introducing pathogens into the wash water and increasing the risk of cross-contamination. The washing process generally involves using chemical sanitizers such as chlorine to minimize cross-contamination; however, the use of sanitizers is not a requirement of FSMA PSR and does not eliminate the total risk of microbial contamination [17]. Research has shown that organic matter and soil can reduce the effectiveness of chlorine-based sanitizers, which is why it is suggested that soil and organic matter be removed from produce before it is washed [18]. 

There is limited information available about the incidence and survival of pathogens in fresh, minimally processed, and frozen strawberries and romaine lettuce. This study focuses on determining the survival rate of *E. coli* on washed romaine lettuce and strawberries. This study will help understand the survival rate of *E. coli* in the washed produce under the commercial supply chain conditions from farm to grocery store, storage, and retail display. The study will also give information about the attachment of *E. coli* to different produce surfaces, including the different layers of lettuce and the leaves and fruit body of strawberries, which marks a research gap in the literature and adds novelty to this study. The potential risk from using contaminated water is the critical treatment parameter evaluated in this study. 

## 2. Materials and Methods

### 2.1. Fresh Produce

Freshly harvested romaine lettuce heads and strawberries were procured (Fresh Produce Distributors, Des Moines, IA, USA) immediately before the treatment to mimic the washing of freshly harvested produce. 

### 2.2. Bacterial Strain and Culture Conditions

#### Rifampicin-Resistant *E. coli*

Microorganisms are naturally found on fresh produce. To account for background flora, rifampicin-resistant *E. coli* O1:K1:H7 (ATCC 11775) was used as an inoculant. The *E. coli* strain was taken from a −80 °C freezer and grown in a tryptic soy broth (TSB) for 24 h at 37 °C. The grown culture was then transferred to rifampicin-tryptic soy agar (RIF-TSA) plates, with a consecutive increase by 20 µg/mL rifampicin each day up to 80 µg/mL [19]. The rifampicin-resistant growth culture was then sub-cultured twice into 80 µg/mL RIF-TSB broth tubes to grow. The final concentration of the *E. coli* cells in the culture was determined to be around 10^9^ log CFU/mL [20]. 

### 2.3. Inoculation and Treatment

**Figure 1:**
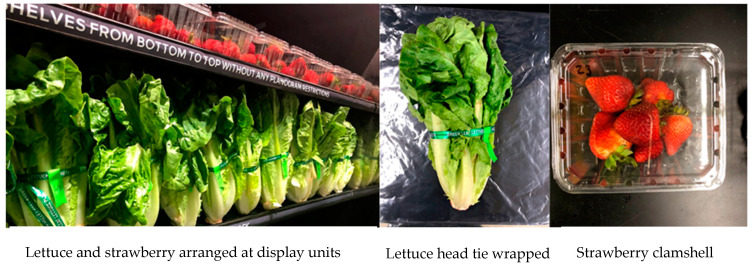
Packaging and storage of the lettuce and strawberries onto display units.

The produce (strawberries or romaine lettuce heads) was dipped and submerged in contaminated water for 1 min with a known concentration of rifampicin-resistant (50 µg/mL) *E. coli* of approximately 10^6^ CFU/mL to mimic industry rinsing practices. After washing, the produce was air-dried for 15 min. Lettuce heads were tie-wrapped and stored in produce crates in an upright position as displayed in the grocery stores. Six strawberries were put into commercially used clamshells and stacked in the produce crates. The packed produce was stored at 4 °C in the walk-in refrigerator for 18 h to mimic long-distance transportation in refrigerated semi-trucks and handling in storage rooms before display. Lettuce and strawberries were displayed on refrigerated shelves (Model VNRBH, Hillphoenix, Conyers, GA, USA) (Figure 1) for 7–8 days of the shelf life period. Day 0 represents the day of washing the produce, day 1 is the transportation time from field to grocery/retail store (24 h), and days 2–8 represent the time mimicking the display at retail stores. Three lettuce bunches and three strawberry clamshells were sampled from the display units for sampling every day. Three independent replications of each treatment (sampling time) were included in the experiment.

### 2.4. Sampling Method

**Figure 2:**
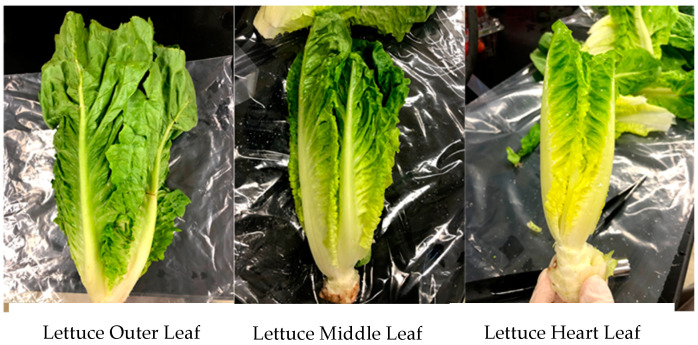
Sampling locations for the outer, middle, and heart leaves of romaine lettuce.

**Figure 3:**
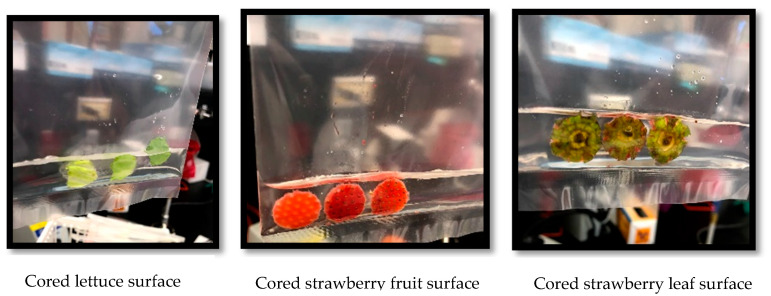
Harvesting cells from the inoculated fruit and leaf surfaces of strawberries.

For lettuces, three random heads (pseudo-replications) were sampled immediately after washing, before putting the produce in storage, and each of the following 7 days (estimated shelf life of lettuce). Each head was sampled using the fruit core (area = 2 cm^2^) in three exterior leaves, three middle leaves, and three heart leaves (Figure 2). The positions sampled within the leaves were the top, center, and lower sections for the exterior, middle, and heart leaves. The three cored samples for each lettuce head position (exterior, middle, and heart) were transferred to a whirlpak containing 10 mL of 0.1% BPW and rubbed manually to transfer the *E. coli* cells (Figure 3). 

For strawberries, a similar sampling technique was used. Three of the six strawberries in each clamshell were cored. The surface layer of the sample was sliced with a knife, put into a whirlpak bag containing 10 mL of 0.1% BPW, and rubbed manually to disperse the microorganisms, representing the fruit sample. The other three strawberries were used to obtain the core from the leaf portions and were put into a single 10 mL of 0.1% BPW and rubbed manually to disperse the microorganisms representing the leaf samples. The weight of the lettuce heads and strawberry clamshells was measured before and after the treatment to determine the water loss over time. 

All samples had a total sampled area of 6 cm^2^ (3 × 2 cm^2^) dispersed in 10 mL of 0.1% BPW. The decimal dilutions were made from the rubbed samples in 0.1% BPW and plated on 50 mg/L Rifampicin TSA plates so that only the rifampicin-resistant *E. coli* could be recovered. In addition, 1 mL of the original sample solution was added to 50 mg/L rifampicin TSB tubes to verify true zeroes if the TSA plates showed no growth after 24 h. 

### 2.5. Statistical Analysis

For the strawberry experiment, the design was a 2 × 7 factorial completely randomized with three replications. Similarly for the lettuce experiment, the design was a 3 × 8 factorial completely randomized with three replications. In both experiments, the first factor was the days of shelf life against the bacterial contamination and the second factor was the variation among the different layers of the produce. The data were analyzed with a two-way ANOVA by using a PROC Glimmix model in SAS (version 9.4). The bacterial counts were log-transformed to meet the assumptions of the ANOVA analysis. The differences in the bacterial survival population among the sampling days and the sampling positions were evaluated using least square means adjustment for multiple comparisons using Tukey’s test. The statistical interpretations were made at a *p* = 0.05 level of confidence. 

## 3. Results

**Figure 4:**
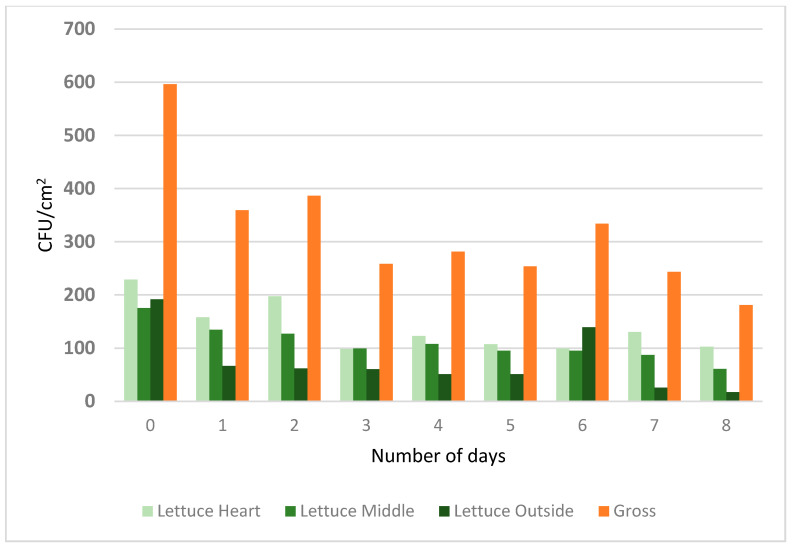
The *E. coli* counts (colony forming units/area) in different layers of romaine lettuce. The letters identify the treatments that are significantly different (*p* < 0.05).

For the lettuce study, the survival of *E. coli* was observed on sampling days for up to a week, with an initial concentration on day 0 of 2.28 log CFU/cm^2^ to 1.64 log CFU/cm^2^ on day 8. The only significant difference found in the bacterial population was between day 0 and day 8 (*p* < 0.001) (see Supplementary Information). With regards to position, the lettuce heart (see Figure 4) had the highest *E. coli* population (2.10 log CFU/cm^2^), followed by the lettuce middle (1.99 log CFU/cm^2^), and the lettuce outer leaf (1.67 log CFU/cm^2^). The heart and middle positions were statistically indifferent (*p* > 0.05). However, compared to the lettuce outer leaf, both these positions reported statistically significant values (*p* > 0.05); see Figure 4 and Supplementary Information. The contamination of lettuce can occur at any point during the production, harvesting, processing, and packaging operations [21]. Moreover, the washing of lettuce heads may not reduce human pathogens [22], but research has shown the likelihood of cross-contamination to non-pathogen-containing lettuce leaves is high [19]. Within this study, the survival of human enteric pathogens in different layers of the lettuce head, i.e., outer, middle, and heart leaves, was observed and showed the importance of the specific location of product sampling when detecting *E. coli*.

**Figure 5:**
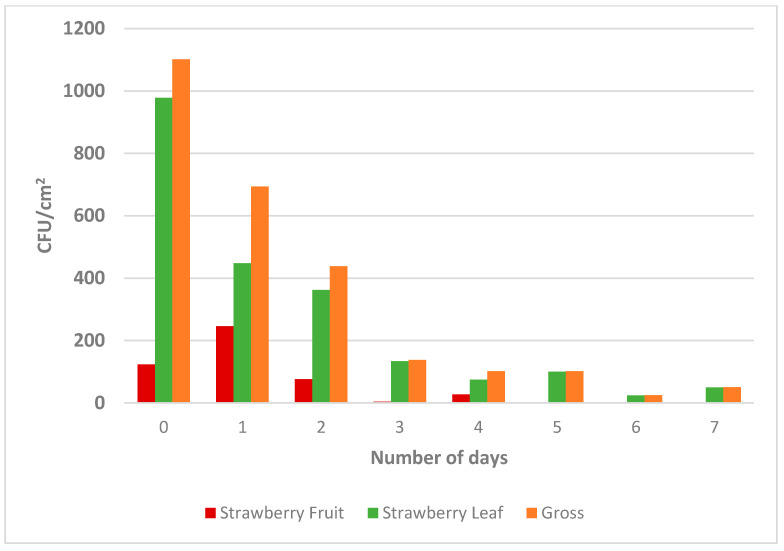
The survival of *E. coli* (colony forming units/area) on Strawberry over 7-Day shelf life. The letters identify the treatments that are significantly different (*p* < 0.05).

For the strawberry study, the survival of *E. coli* was observed on sampling days up to seven days, with an initial concentration on day 0 of 3.0 log CFU/cm^2^ to 1.7 log CFU/cm^2^ on day 7 (Figure 5). The bacterial population was significantly different between days 0, 1, and 2 (*p* < 0.001) (see Supplementary Information). With regards to position, the strawberry leaf had a higher *E. coli* population (1.62 log CFU/cm^2^) compared to the strawberry fruit (0.29 log CFU/cm^2^), the statistical difference between which was significant (*p* < 0.001). These findings provide significant evidence on the contamination potential of strawberry leaves and may lead to the development of best practices such as removing strawberry leaves for fresh-cut applications and fruit salads where leaves are generally not removed from the fruit to extend the shelf life. Additionally, strawberries are typically hand-picked by the leaf and stem area. If this area is the primary source of contamination, hand hygiene is essential throughout the harvesting shift. This research also highlights the importance of testing the leaves as many strawberry products are presented to the consumer with an entire stem attached. Several studies have reported the survival of foodborne bacteria on the strawberries, and to date, no study has evaluated the differences between survival on the fruit and the leaves of strawberries. One study reported the survival of *E. coli* on strawberry plants during growing under greenhouses conditions and reported cross-contamination from the leaves to the fruit with the presence of *E. coli* on uninoculated strawberries [23]. Another study investigated the survival of *E. coli* inside and outside the fruit and reported a reduction in the pathogen population using different chemical agents [24].

Similarly, another paper reported the attachment and survival of 13 different foodborne bacteria on strawberries and investigated the effects of different antimicrobial treatments [25]. Several studies observed that many of the bacterial species can survive for 15 days [23,25]. Both *E. coli* and *Salmonella* have been reported to survive for longer than one month on frozen strawberries [26]. 

Fresh produce is essential to a well-balanced, healthy diet. According to myplate.gov (accessed on 1 January 2021), one-half of every meal should be consumed with fruits and vegetables [27]. It is important to handle fresh produce using Good Agricultural Practices (GAP) to ensure the safety of harmful pathogens. Contamination is highest during three periods: in the field, during initial processing, and in the final preparation stage in the kitchen. During the process, for instance, animal livestock manure or wildlife are present in the field. The rain or overhead vegetation can cause runoff contamination, mainly to produce grown on the ground such as lettuce and strawberries. 

Considering the significant food safety risk associated with the minimal processing of strawberries, and knowing that field-grown strawberries can have opportunistic human pathogens [28], research efforts have been made to investigate the effect of various wash methods and technologies to lower the human pathogen load on the berries. A study by the authors in [29] reported the potential application of an antimicrobial wash, an antimicrobial coating, and a combination of both, reporting antimicrobial effectiveness and the preservation of the berries’ color, texture, and appearance. Another study reported an increased inactivation of 13 foodborne bacteria using roselle calyx extracts that performed better than other commonly used chemical methods in the produce wash industry i.e., chlorination and organic acids [25]. Novel non-thermal technologies such as ultraviolet light and water-assisted pulsed light have also been investigated and have reported significant efficacy against foodborne pathogen reduction in berries [30]. 

## 4. Conclusions

This study highlights the importance of using safe wash water and adequate sanitary quality to protect the microbial safety of fresh produce while protecting public health. The results indicated that E. coli can survive in both lettuce and strawberries over extended periods. A survival population of 2.3 log CFU/cm^2^ (day 8) was observed on lettuce with an initial population of 2.8 log CFU/cm^2^ (day 0). On strawberries, the population reduced from 3.0 (day 0) to 1.7 log CFU/cm^2^ (day 7), with an initial *E. coli* concentration of approx. 6 log CFU/mL in the wash water. Strawberry leaves had a higher attachment of *E. coli* than the fruit (*p* < 0.05). Additionally, this study suggests the importance of sampling leafy greens within the core of the product and not only the outer layers.

Also, this study suggested that sampling some of the strawberry leaves in addition to the strawberry fruit is important. Further studies are needed to investigate the survival of pathogens in different layers of lettuce using advanced technologies such as 3D- Surface plots, etc. The conclusions from these advanced studies focusing on the survival of human pathogens in storage conditions of fresh produce might lead to the development of best practices for displaying and storing fresh produce in different retail settings that will minimize the contamination risk. 

## Supplementary Materials

The following are available online at https://www.mdpi.com/article/10.3390/foods10061390/s1, Supplementary Information: Data Analysis for Produce Wash Study–July 2020.
