# Supplementary material for: Escherichia coli Survival on Strawberries and Unpacked Romaine Lettuce Washed Using Contaminated Water
Source: Foods. 2021 Jun 16;10(6):1390. doi: 10.3390/foods10061390 (PMC8235505; doi:10.3390/foods10061390)
Supplement: Supplementary file 1 [file foods-10-01390-s001.zip › foods-1239102-supplementary.pdf]

Data Analysis for Produce Wash Study – July 2020

Lettuce Data

The SAS System

The GLIMMIX Procedure

Model Information	
**Data Set**	WORK.PWS
**Response Variable**	log
**Response Distribution**	Gaussian
**Link Function**	Identity
**Variance Function**	Default
**Variance Matrix**	Not blocked
**Estimation Technique**	Restricted Maximum Likelihood
**Degrees of Freedom Method**	Containment

Class Level Information		
**Class**	**Levels**	**Values**
**Position**	3	Heart Middle Out
**Day**	9	-1 0 1 2 3 4 5 6 7
**Rep**	3	1 2 3

**Number of Observations Read**	81
**Number of Observations Used**	81

Dimensions	
**G-side Cov. Parameters**	2
**R-side Cov. Parameters**	1
**Columns in X**	43
**Columns in Z**	3
**Subjects (Blocks in V)**	1
**Max Obs per Subject**	81

Optimization Information

**Optimization Technique**	Dual Quasi-Newton
**Parameters in Optimization**	2
**Lower Boundaries**	1
**Upper Boundaries**	0
**Fixed Effects**	Profiled
**Residual Variance**	Profiled
**Starting From**	Data

Iteration History					
**Iteration**	**Restarts**	**Evaluations**	**Objective Function**	**Change**	**Max Gradient**
**0**	**0**	4	18.326538398	.	0

Convergence criterion (ABSGCONV=0.00001) satisfied.

Estimated G matrix is not positive definite.

Fit Statistics	
**-2 Res Log Likelihood**	18.33
**AIC (smaller is better)**	22.33
**AICC (smaller is better)**	22.57
**BIC (smaller is better)**	20.52
**CAIC (smaller is better)**	22.52
**HQIC (smaller is better)**	18.70
**Generalized Chi-Square**	2.20
**Gener. Chi-Square / DF**	0.04

Covariance Parameter Estimates		
**Cov Parm**	**Estimate**	**Standard Error**
**Variance**	0	.
**CS**	0	.
**Residual**	0.04236	0.008308

Type III Tests of Fixed Effects				
**Effect**	**Num DF**	**Den DF**	**F Value**	**Pr > F**
**Position**	2	52	31.35	<.0001
**Day**	8	52	6.75	<.0001
**Position*Day**	16	52	1.02	0.4564
**Rep**	2	0	0.96	.

Position Least Squares Means					
**Position**	**Estimate**	**Standard Error**	**DF**	**t Value**	**Pr > |t|**
**Heart**	2.1034	0.03961	52	53.10	<.0001
**Middle**	1.9851	0.03961	52	50.12	<.0001
**Out**	1.6740	0.03961	52	42.26	<.0001

Differences of Position Least Squares Means<br>Adjustment for Multiple Comparisons: Tukey							
**Position**	**_Position**	**Estimate**	**Standard Error**	**DF**	**t Value**	**Pr > |t|**	**Adj P**
**Heart**	**Middle**	0.1183	0.05602	52	2.11	0.0395	0.0971
**Heart**	**Out**	0.4294	0.05602	52	7.67	<.0001	<.0001
**Middle**	**Out**	0.3110	0.05602	52	5.55	<.0001	<.0001

Tukey Grouping for Position Least Squares Means (Alpha=0.05)		
**LS-means with the same letter are not significantly different.**		
**Position**	**Estimate**	
**Heart**	2.1034	A
		A
**Middle**	1.9851	A
**Out**	1.6740	B

Day Least Squares Means

Day	Estimate	Standard Error	DF	t Value	Pr > |t|
**-1**	2.2858	0.06861	52	33.32	<.0001
**0**	2.0310	0.06861	52	29.60	<.0001
**1**	1.9944	0.06861	52	29.07	<.0001
**2**	1.8936	0.06861	52	27.60	<.0001
**3**	1.9200	0.06861	52	27.99	<.0001
**4**	1.8797	0.06861	52	27.40	<.0001
**5**	1.8591	0.06861	52	27.10	<.0001
**6**	1.7828	0.06861	52	25.99	<.0001
**7**	1.6412	0.06861	52	23.92	<.0001

Differences of Day Least Squares Means
Adjustment for Multiple Comparisons: Tukey

Day	_Day	Estimate	Standard Error	DF	t Value	Pr > |t|	Adj P
**-1**	**0**	0.2548	0.09702	52	2.63	0.0113	0.2011
**-1**	**1**	0.2913	0.09702	52	3.00	0.0041	0.0887
**-1**	**2**	0.3922	0.09702	52	4.04	0.0002	0.0051
**-1**	**3**	0.3658	0.09702	52	3.77	0.0004	0.0115
**-1**	**4**	0.4061	0.09702	52	4.19	0.0001	0.0033
**-1**	**5**	0.4267	0.09702	52	4.40	<.0001	0.0017
**-1**	**6**	0.5030	0.09702	52	5.18	<.0001	0.0001
**-1**	**7**	0.6446	0.09702	52	6.64	<.0001	<.0001
**0**	**1**	0.03656	0.09702	52	0.38	0.7079	1.0000
**0**	**2**	0.1374	0.09702	52	1.42	0.1626	0.8863
**0**	**3**	0.1110	0.09702	52	1.14	0.2578	0.9643
**0**	**4**	0.1513	0.09702	52	1.56	0.1249	0.8214
**0**	**5**	0.1719	0.09702	52	1.77	0.0823	0.6999
**0**	**6**	0.2482	0.09702	52	2.56	0.0135	0.2294
**0**	**7**	0.3898	0.09702	52	4.02	0.0002	0.0055
**1**	**2**	0.1009	0.09702	52	1.04	0.3032	0.9799
**1**	**3**	0.07444	0.09702	52	0.77	0.4464	0.9973
**1**	**4**	0.1148	0.09702	52	1.18	0.2422	0.9566
**1**	**5**	0.1353	0.09702	52	1.39	0.1690	0.8947

**1**	**6**	0.2117	0.09702	52	2.18	0.0337	0.4324
**1**	**7**	0.3532	0.09702	52	3.64	0.0006	0.0167
**2**	**3**	-0.02644	0.09702	52	-0.27	0.7863	1.0000
**2**	**4**	0.01389	0.09702	52	0.14	0.8867	1.0000
**2**	**5**	0.03444	0.09702	52	0.36	0.7240	1.0000
**2**	**6**	0.1108	0.09702	52	1.14	0.2588	0.9647
**2**	**7**	0.2523	0.09702	52	2.60	0.0121	0.2114
**3**	**4**	0.04033	0.09702	52	0.42	0.6793	1.0000
**3**	**5**	0.06089	0.09702	52	0.63	0.5330	0.9994
**3**	**6**	0.1372	0.09702	52	1.41	0.1632	0.8872
**3**	**7**	0.2788	0.09702	52	2.87	0.0059	0.1193
**4**	**5**	0.02056	0.09702	52	0.21	0.8330	1.0000
**4**	**6**	0.09689	0.09702	52	1.00	0.3226	0.9844
**4**	**7**	0.2384	0.09702	52	2.46	0.0174	0.2763
**5**	**6**	0.07633	0.09702	52	0.79	0.4350	0.9968
**5**	**7**	0.2179	0.09702	52	2.25	0.0290	0.3930
**6**	**7**	0.1416	0.09702	52	1.46	0.1506	0.8688

**Tukey Grouping for Day Least Squares Means (Alpha=0.05)**			
**LS-means with the same letter are not significantly different.**			
**Day**	**Estimate**		
**-1**	2.2858		A
			A
**0**	2.0310	B	A
		B	A
**1**	1.9944	B	A
		B	
**3**	1.9200	B	C
		B	C
**2**	1.8936	B	C
		B	C

**4**	1.8797	B	C
		B	C
**5**	1.8591	B	C
		B	C
**6**	1.7828	B	C
			C
**7**	1.6412		C

**Position*Day Least Squares Means**						
**Position**	**Day**	**Estimate**	**Standard Error**	**DF**	**t Value**	**Pr > |t|**
**Heart**	**-1**	2.3493	0.1188	52	19.77	<.0001
**Heart**	**0**	2.1870	0.1188	52	18.40	<.0001
**Heart**	**1**	2.2377	0.1188	52	18.83	<.0001
**Heart**	**2**	1.9797	0.1188	52	16.66	<.0001
**Heart**	**3**	2.0800	0.1188	52	17.50	<.0001
**Heart**	**4**	2.0280	0.1188	52	17.07	<.0001
**Heart**	**5**	1.9720	0.1188	52	16.60	<.0001
**Heart**	**6**	2.0993	0.1188	52	17.67	<.0001
**Heart**	**7**	1.9977	0.1188	52	16.81	<.0001
**Middle**	**-1**	2.2400	0.1188	52	18.85	<.0001
**Middle**	**0**	2.1280	0.1188	52	17.91	<.0001
**Middle**	**1**	1.9923	0.1188	52	16.77	<.0001
**Middle**	**2**	1.9747	0.1188	52	16.62	<.0001
**Middle**	**3**	2.0227	0.1188	52	17.02	<.0001
**Middle**	**4**	1.9580	0.1188	52	16.48	<.0001
**Middle**	**5**	1.9560	0.1188	52	16.46	<.0001
**Middle**	**6**	1.8500	0.1188	52	15.57	<.0001
**Middle**	**7**	1.7440	0.1188	52	14.68	<.0001
**Out**	**-1**	2.2680	0.1188	52	19.09	<.0001
**Out**	**0**	1.7780	0.1188	52	14.96	<.0001
**Out**	**1**	1.7533	0.1188	52	14.75	<.0001
**Out**	**2**	1.7263	0.1188	52	14.53	<.0001
**Out**	**3**	1.6573	0.1188	52	13.95	<.0001

**Out**	**4**	1.6530	0.1188	52	13.91	<.0001
**Out**	**5**	1.6493	0.1188	52	13.88	<.0001
**Out**	**6**	1.3990	0.1188	52	11.77	<.0001
**Out**	**7**	1.1820	0.1188	52	9.95	<.0001

Differences of Position*Day Least Squares Means
Adjustment for Multiple Comparisons: Tukey

Position	Day	_Position	_Day	Estimate	Standard Error	DF	t Value	Pr > |t|	Adj P
**Heart**	**-1**	**Heart**	**0**	0.1623	0.1681	52	0.97	0.3385	1.0000
**Heart**	**-1**	**Heart**	**1**	0.1117	0.1681	52	0.66	0.5093	1.0000
**Heart**	**-1**	**Heart**	**2**	0.3697	0.1681	52	2.20	0.0323	0.8870
**Heart**	**-1**	**Heart**	**3**	0.2693	0.1681	52	1.60	0.1151	0.9966
**Heart**	**-1**	**Heart**	**4**	0.3213	0.1681	52	1.91	0.0614	0.9702
**Heart**	**-1**	**Heart**	**5**	0.3773	0.1681	52	2.25	0.0290	0.8668
**Heart**	**-1**	**Heart**	**6**	0.2500	0.1681	52	1.49	0.1429	0.9988
**Heart**	**-1**	**Heart**	**7**	0.3517	0.1681	52	2.09	0.0413	0.9268
**Heart**	**-1**	**Middle**	**-1**	0.1093	0.1681	52	0.65	0.5182	1.0000
**Heart**	**-1**	**Middle**	**0**	0.2213	0.1681	52	1.32	0.1936	0.9998
**Heart**	**-1**	**Middle**	**1**	0.3570	0.1681	52	2.12	0.0384	0.9161
**Heart**	**-1**	**Middle**	**2**	0.3747	0.1681	52	2.23	0.0301	0.8740
**Heart**	**-1**	**Middle**	**3**	0.3267	0.1681	52	1.94	0.0573	0.9645
**Heart**	**-1**	**Middle**	**4**	0.3913	0.1681	52	2.33	0.0238	0.8248
**Heart**	**-1**	**Middle**	**5**	0.3933	0.1681	52	2.34	0.0231	0.8183
**Heart**	**-1**	**Middle**	**6**	0.4993	0.1681	52	2.97	0.0045	0.3937
**Heart**	**-1**	**Middle**	**7**	0.6053	0.1681	52	3.60	0.0007	0.1083
**Heart**	**-1**	**Out**	**-1**	0.08133	0.1681	52	0.48	0.6304	1.0000
**Heart**	**-1**	**Out**	**0**	0.5713	0.1681	52	3.40	0.0013	0.1725
**Heart**	**-1**	**Out**	**1**	0.5960	0.1681	52	3.55	0.0008	0.1235
**Heart**	**-1**	**Out**	**2**	0.6230	0.1681	52	3.71	0.0005	0.0836
**Heart**	**-1**	**Out**	**3**	0.6920	0.1681	52	4.12	0.0001	0.0278
**Heart**	**-1**	**Out**	**4**	0.6963	0.1681	52	4.14	0.0001	0.0258
**Heart**	**-1**	**Out**	**5**	0.7000	0.1681	52	4.17	0.0001	0.0243
**Heart**	**-1**	**Out**	**6**	0.9503	0.1681	52	5.66	<.0001	0.0002
**Heart**	**-1**	**Out**	**7**	1.1673	0.1681	52	6.95	<.0001	<.0001

**Heart**	**0**	**Heart**	**1**	-0.05067	0.1681	52	-0.30	0.7642	1.0000
**Heart**	**0**	**Heart**	**2**	0.2073	0.1681	52	1.23	0.2228	0.9999
**Heart**	**0**	**Heart**	**3**	0.1070	0.1681	52	0.64	0.5271	1.0000
**Heart**	**0**	**Heart**	**4**	0.1590	0.1681	52	0.95	0.3485	1.0000
**Heart**	**0**	**Heart**	**5**	0.2150	0.1681	52	1.28	0.2064	0.9999
**Heart**	**0**	**Heart**	**6**	0.08767	0.1681	52	0.52	0.6041	1.0000
**Heart**	**0**	**Heart**	**7**	0.1893	0.1681	52	1.13	0.2651	1.0000
**Heart**	**0**	**Middle**	**-1**	-0.05300	0.1681	52	-0.32	0.7537	1.0000
**Heart**	**0**	**Middle**	**0**	0.05900	0.1681	52	0.35	0.7269	1.0000
**Heart**	**0**	**Middle**	**1**	0.1947	0.1681	52	1.16	0.2520	1.0000
**Heart**	**0**	**Middle**	**2**	0.2123	0.1681	52	1.26	0.2120	0.9999
**Heart**	**0**	**Middle**	**3**	0.1643	0.1681	52	0.98	0.3327	1.0000
**Heart**	**0**	**Middle**	**4**	0.2290	0.1681	52	1.36	0.1789	0.9997
**Heart**	**0**	**Middle**	**5**	0.2310	0.1681	52	1.37	0.1752	0.9997
**Heart**	**0**	**Middle**	**6**	0.3370	0.1681	52	2.01	0.0501	0.9512
**Heart**	**0**	**Middle**	**7**	0.4430	0.1681	52	2.64	0.0110	0.6273
**Heart**	**0**	**Out**	**-1**	-0.08100	0.1681	52	-0.48	0.6318	1.0000
**Heart**	**0**	**Out**	**0**	0.4090	0.1681	52	2.43	0.0184	0.7636
**Heart**	**0**	**Out**	**1**	0.4337	0.1681	52	2.58	0.0127	0.6663
**Heart**	**0**	**Out**	**2**	0.4607	0.1681	52	2.74	0.0084	0.5522
**Heart**	**0**	**Out**	**3**	0.5297	0.1681	52	3.15	0.0027	0.2863
**Heart**	**0**	**Out**	**4**	0.5340	0.1681	52	3.18	0.0025	0.2725
**Heart**	**0**	**Out**	**5**	0.5377	0.1681	52	3.20	0.0023	0.2613
**Heart**	**0**	**Out**	**6**	0.7880	0.1681	52	4.69	<.0001	0.0050
**Heart**	**0**	**Out**	**7**	1.0050	0.1681	52	5.98	<.0001	<.0001
**Heart**	**1**	**Heart**	**2**	0.2580	0.1681	52	1.54	0.1308	0.9982
**Heart**	**1**	**Heart**	**3**	0.1577	0.1681	52	0.94	0.3525	1.0000
**Heart**	**1**	**Heart**	**4**	0.2097	0.1681	52	1.25	0.2178	0.9999
**Heart**	**1**	**Heart**	**5**	0.2657	0.1681	52	1.58	0.1200	0.9972
**Heart**	**1**	**Heart**	**6**	0.1383	0.1681	52	0.82	0.4142	1.0000
**Heart**	**1**	**Heart**	**7**	0.2400	0.1681	52	1.43	0.1592	0.9994
**Heart**	**1**	**Middle**	**-1**	-0.00233	0.1681	52	-0.01	0.9890	1.0000
**Heart**	**1**	**Middle**	**0**	0.1097	0.1681	52	0.65	0.5169	1.0000

**Heart**	**1**	**Middle**	**1**	0.2453	0.1681	52	1.46	0.1503	0.9991
**Heart**	**1**	**Middle**	**2**	0.2630	0.1681	52	1.57	0.1236	0.9976
**Heart**	**1**	**Middle**	**3**	0.2150	0.1681	52	1.28	0.2064	0.9999
**Heart**	**1**	**Middle**	**4**	0.2797	0.1681	52	1.66	0.1021	0.9944
**Heart**	**1**	**Middle**	**5**	0.2817	0.1681	52	1.68	0.0997	0.9938
**Heart**	**1**	**Middle**	**6**	0.3877	0.1681	52	2.31	0.0251	0.8364
**Heart**	**1**	**Middle**	**7**	0.4937	0.1681	52	2.94	0.0049	0.4158
**Heart**	**1**	**Out**	**-1**	-0.03033	0.1681	52	-0.18	0.8575	1.0000
**Heart**	**1**	**Out**	**0**	0.4597	0.1681	52	2.74	0.0085	0.5564
**Heart**	**1**	**Out**	**1**	0.4843	0.1681	52	2.88	0.0057	0.4532
**Heart**	**1**	**Out**	**2**	0.5113	0.1681	52	3.04	0.0037	0.3490
**Heart**	**1**	**Out**	**3**	0.5803	0.1681	52	3.45	0.0011	0.1531
**Heart**	**1**	**Out**	**4**	0.5847	0.1681	52	3.48	0.0010	0.1444
**Heart**	**1**	**Out**	**5**	0.5883	0.1681	52	3.50	0.0010	0.1374
**Heart**	**1**	**Out**	**6**	0.8387	0.1681	52	4.99	<.0001	0.0019
**Heart**	**1**	**Out**	**7**	1.0557	0.1681	52	6.28	<.0001	<.0001
**Heart**	**2**	**Heart**	**3**	-0.1003	0.1681	52	-0.60	0.5531	1.0000
**Heart**	**2**	**Heart**	**4**	-0.04833	0.1681	52	-0.29	0.7748	1.0000
**Heart**	**2**	**Heart**	**5**	0.007667	0.1681	52	0.05	0.9638	1.0000
**Heart**	**2**	**Heart**	**6**	-0.1197	0.1681	52	-0.71	0.4796	1.0000
**Heart**	**2**	**Heart**	**7**	-0.01800	0.1681	52	-0.11	0.9151	1.0000
**Heart**	**2**	**Middle**	**-1**	-0.2603	0.1681	52	-1.55	0.1274	0.9979
**Heart**	**2**	**Middle**	**0**	-0.1483	0.1681	52	-0.88	0.3815	1.0000
**Heart**	**2**	**Middle**	**1**	-0.01267	0.1681	52	-0.08	0.9402	1.0000
**Heart**	**2**	**Middle**	**2**	0.005000	0.1681	52	0.03	0.9764	1.0000
**Heart**	**2**	**Middle**	**3**	-0.04300	0.1681	52	-0.26	0.7991	1.0000
**Heart**	**2**	**Middle**	**4**	0.02167	0.1681	52	0.13	0.8979	1.0000
**Heart**	**2**	**Middle**	**5**	0.02367	0.1681	52	0.14	0.8885	1.0000
**Heart**	**2**	**Middle**	**6**	0.1297	0.1681	52	0.77	0.4438	1.0000
**Heart**	**2**	**Middle**	**7**	0.2357	0.1681	52	1.40	0.1668	0.9995
**Heart**	**2**	**Out**	**-1**	-0.2883	0.1681	52	-1.72	0.0922	0.9917
**Heart**	**2**	**Out**	**0**	0.2017	0.1681	52	1.20	0.2356	1.0000
**Heart**	**2**	**Out**	**1**	0.2263	0.1681	52	1.35	0.1839	0.9998

**Heart**	**2**	**Out**	**2**	0.2533	0.1681	52	1.51	0.1377	0.9986
**Heart**	**2**	**Out**	**3**	0.3223	0.1681	52	1.92	0.0606	0.9692
**Heart**	**2**	**Out**	**4**	0.3267	0.1681	52	1.94	0.0573	0.9645
**Heart**	**2**	**Out**	**5**	0.3303	0.1681	52	1.97	0.0547	0.9601
**Heart**	**2**	**Out**	**6**	0.5807	0.1681	52	3.46	0.0011	0.1524
**Heart**	**2**	**Out**	**7**	0.7977	0.1681	52	4.75	<.0001	0.0041
**Heart**	**3**	**Heart**	**4**	0.05200	0.1681	52	0.31	0.7582	1.0000
**Heart**	**3**	**Heart**	**5**	0.1080	0.1681	52	0.64	0.5233	1.0000
**Heart**	**3**	**Heart**	**6**	-0.01933	0.1681	52	-0.12	0.9089	1.0000
**Heart**	**3**	**Heart**	**7**	0.08233	0.1681	52	0.49	0.6262	1.0000
**Heart**	**3**	**Middle**	**-1**	-0.1600	0.1681	52	-0.95	0.3455	1.0000
**Heart**	**3**	**Middle**	**0**	-0.04800	0.1681	52	-0.29	0.7763	1.0000
**Heart**	**3**	**Middle**	**1**	0.08767	0.1681	52	0.52	0.6041	1.0000
**Heart**	**3**	**Middle**	**2**	0.1053	0.1681	52	0.63	0.5335	1.0000
**Heart**	**3**	**Middle**	**3**	0.05733	0.1681	52	0.34	0.7344	1.0000
**Heart**	**3**	**Middle**	**4**	0.1220	0.1681	52	0.73	0.4711	1.0000
**Heart**	**3**	**Middle**	**5**	0.1240	0.1681	52	0.74	0.4639	1.0000
**Heart**	**3**	**Middle**	**6**	0.2300	0.1681	52	1.37	0.1770	0.9997
**Heart**	**3**	**Middle**	**7**	0.3360	0.1681	52	2.00	0.0508	0.9526
**Heart**	**3**	**Out**	**-1**	-0.1880	0.1681	52	-1.12	0.2684	1.0000
**Heart**	**3**	**Out**	**0**	0.3020	0.1681	52	1.80	0.0781	0.9852
**Heart**	**3**	**Out**	**1**	0.3267	0.1681	52	1.94	0.0573	0.9645
**Heart**	**3**	**Out**	**2**	0.3537	0.1681	52	2.10	0.0402	0.9229
**Heart**	**3**	**Out**	**3**	0.4227	0.1681	52	2.52	0.0150	0.7110
**Heart**	**3**	**Out**	**4**	0.4270	0.1681	52	2.54	0.0141	0.6936
**Heart**	**3**	**Out**	**5**	0.4307	0.1681	52	2.56	0.0133	0.6787
**Heart**	**3**	**Out**	**6**	0.6810	0.1681	52	4.05	0.0002	0.0334
**Heart**	**3**	**Out**	**7**	0.8980	0.1681	52	5.34	<.0001	0.0006
**Heart**	**4**	**Heart**	**5**	0.05600	0.1681	52	0.33	0.7403	1.0000
**Heart**	**4**	**Heart**	**6**	-0.07133	0.1681	52	-0.42	0.6730	1.0000
**Heart**	**4**	**Heart**	**7**	0.03033	0.1681	52	0.18	0.8575	1.0000
**Heart**	**4**	**Middle**	**-1**	-0.2120	0.1681	52	-1.26	0.2128	0.9999
**Heart**	**4**	**Middle**	**0**	-0.1000	0.1681	52	-0.60	0.5544	1.0000

**Heart**	**4**	**Middle**	**1**	0.03567	0.1681	52	0.21	0.8328	1.0000
**Heart**	**4**	**Middle**	**2**	0.05333	0.1681	52	0.32	0.7522	1.0000
**Heart**	**4**	**Middle**	**3**	0.005333	0.1681	52	0.03	0.9748	1.0000
**Heart**	**4**	**Middle**	**4**	0.07000	0.1681	52	0.42	0.6787	1.0000
**Heart**	**4**	**Middle**	**5**	0.07200	0.1681	52	0.43	0.6701	1.0000
**Heart**	**4**	**Middle**	**6**	0.1780	0.1681	52	1.06	0.2944	1.0000
**Heart**	**4**	**Middle**	**7**	0.2840	0.1681	52	1.69	0.0970	0.9931
**Heart**	**4**	**Out**	**-1**	-0.2400	0.1681	52	-1.43	0.1592	0.9994
**Heart**	**4**	**Out**	**0**	0.2500	0.1681	52	1.49	0.1429	0.9988
**Heart**	**4**	**Out**	**1**	0.2747	0.1681	52	1.63	0.1082	0.9956
**Heart**	**4**	**Out**	**2**	0.3017	0.1681	52	1.80	0.0785	0.9854
**Heart**	**4**	**Out**	**3**	0.3707	0.1681	52	2.21	0.0319	0.8845
**Heart**	**4**	**Out**	**4**	0.3750	0.1681	52	2.23	0.0300	0.8731
**Heart**	**4**	**Out**	**5**	0.3787	0.1681	52	2.25	0.0285	0.8630
**Heart**	**4**	**Out**	**6**	0.6290	0.1681	52	3.74	0.0005	0.0764
**Heart**	**4**	**Out**	**7**	0.8460	0.1681	52	5.03	<.0001	0.0016
**Heart**	**5**	**Heart**	**6**	-0.1273	0.1681	52	-0.76	0.4520	1.0000
**Heart**	**5**	**Heart**	**7**	-0.02567	0.1681	52	-0.15	0.8792	1.0000
**Heart**	**5**	**Middle**	**-1**	-0.2680	0.1681	52	-1.59	0.1168	0.9968
**Heart**	**5**	**Middle**	**0**	-0.1560	0.1681	52	-0.93	0.3575	1.0000
**Heart**	**5**	**Middle**	**1**	-0.02033	0.1681	52	-0.12	0.9042	1.0000
**Heart**	**5**	**Middle**	**2**	-0.00267	0.1681	52	-0.02	0.9874	1.0000
**Heart**	**5**	**Middle**	**3**	-0.05067	0.1681	52	-0.30	0.7642	1.0000
**Heart**	**5**	**Middle**	**4**	0.01400	0.1681	52	0.08	0.9339	1.0000
**Heart**	**5**	**Middle**	**5**	0.01600	0.1681	52	0.10	0.9245	1.0000
**Heart**	**5**	**Middle**	**6**	0.1220	0.1681	52	0.73	0.4711	1.0000
**Heart**	**5**	**Middle**	**7**	0.2280	0.1681	52	1.36	0.1807	0.9997
**Heart**	**5**	**Out**	**-1**	-0.2960	0.1681	52	-1.76	0.0841	0.9884
**Heart**	**5**	**Out**	**0**	0.1940	0.1681	52	1.15	0.2536	1.0000
**Heart**	**5**	**Out**	**1**	0.2187	0.1681	52	1.30	0.1989	0.9999
**Heart**	**5**	**Out**	**2**	0.2457	0.1681	52	1.46	0.1498	0.9991
**Heart**	**5**	**Out**	**3**	0.3147	0.1681	52	1.87	0.0668	0.9763
**Heart**	**5**	**Out**	**4**	0.3190	0.1681	52	1.90	0.0632	0.9724

**Heart**	**5**	**Out**	**5**	0.3227	0.1681	52	1.92	0.0603	0.9688
**Heart**	**5**	**Out**	**6**	0.5730	0.1681	52	3.41	0.0013	0.1687
**Heart**	**5**	**Out**	**7**	0.7900	0.1681	52	4.70	<.0001	0.0048
**Heart**	**6**	**Heart**	**7**	0.1017	0.1681	52	0.60	0.5478	1.0000
**Heart**	**6**	**Middle**	**-1**	-0.1407	0.1681	52	-0.84	0.4064	1.0000
**Heart**	**6**	**Middle**	**0**	-0.02867	0.1681	52	-0.17	0.8652	1.0000
**Heart**	**6**	**Middle**	**1**	0.1070	0.1681	52	0.64	0.5271	1.0000
**Heart**	**6**	**Middle**	**2**	0.1247	0.1681	52	0.74	0.4615	1.0000
**Heart**	**6**	**Middle**	**3**	0.07667	0.1681	52	0.46	0.6501	1.0000
**Heart**	**6**	**Middle**	**4**	0.1413	0.1681	52	0.84	0.4042	1.0000
**Heart**	**6**	**Middle**	**5**	0.1433	0.1681	52	0.85	0.3976	1.0000
**Heart**	**6**	**Middle**	**6**	0.2493	0.1681	52	1.48	0.1439	0.9989
**Heart**	**6**	**Middle**	**7**	0.3553	0.1681	52	2.11	0.0393	0.9195
**Heart**	**6**	**Out**	**-1**	-0.1687	0.1681	52	-1.00	0.3202	1.0000
**Heart**	**6**	**Out**	**0**	0.3213	0.1681	52	1.91	0.0614	0.9702
**Heart**	**6**	**Out**	**1**	0.3460	0.1681	52	2.06	0.0445	0.9370
**Heart**	**6**	**Out**	**2**	0.3730	0.1681	52	2.22	0.0308	0.8785
**Heart**	**6**	**Out**	**3**	0.4420	0.1681	52	2.63	0.0112	0.6315
**Heart**	**6**	**Out**	**4**	0.4463	0.1681	52	2.66	0.0105	0.6132
**Heart**	**6**	**Out**	**5**	0.4500	0.1681	52	2.68	0.0099	0.5976
**Heart**	**6**	**Out**	**6**	0.7003	0.1681	52	4.17	0.0001	0.0241
**Heart**	**6**	**Out**	**7**	0.9173	0.1681	52	5.46	<.0001	0.0004
**Heart**	**7**	**Middle**	**-1**	-0.2423	0.1681	52	-1.44	0.1553	0.9993
**Heart**	**7**	**Middle**	**0**	-0.1303	0.1681	52	-0.78	0.4415	1.0000
**Heart**	**7**	**Middle**	**1**	0.005333	0.1681	52	0.03	0.9748	1.0000
**Heart**	**7**	**Middle**	**2**	0.02300	0.1681	52	0.14	0.8917	1.0000
**Heart**	**7**	**Middle**	**3**	-0.02500	0.1681	52	-0.15	0.8823	1.0000
**Heart**	**7**	**Middle**	**4**	0.03967	0.1681	52	0.24	0.8143	1.0000
**Heart**	**7**	**Middle**	**5**	0.04167	0.1681	52	0.25	0.8052	1.0000
**Heart**	**7**	**Middle**	**6**	0.1477	0.1681	52	0.88	0.3836	1.0000
**Heart**	**7**	**Middle**	**7**	0.2537	0.1681	52	1.51	0.1372	0.9986
**Heart**	**7**	**Out**	**-1**	-0.2703	0.1681	52	-1.61	0.1138	0.9964
**Heart**	**7**	**Out**	**0**	0.2197	0.1681	52	1.31	0.1969	0.9999

**Heart**	**7**	**Out**	**1**	0.2443	0.1681	52	1.45	0.1520	0.9992
**Heart**	**7**	**Out**	**2**	0.2713	0.1681	52	1.61	0.1125	0.9963
**Heart**	**7**	**Out**	**3**	0.3403	0.1681	52	2.03	0.0480	0.9462
**Heart**	**7**	**Out**	**4**	0.3447	0.1681	52	2.05	0.0453	0.9393
**Heart**	**7**	**Out**	**5**	0.3483	0.1681	52	2.07	0.0432	0.9329
**Heart**	**7**	**Out**	**6**	0.5987	0.1681	52	3.56	0.0008	0.1190
**Heart**	**7**	**Out**	**7**	0.8157	0.1681	52	4.85	<.0001	0.0029
**Middle**	**-1**	**Middle**	**0**	0.1120	0.1681	52	0.67	0.5081	1.0000
**Middle**	**-1**	**Middle**	**1**	0.2477	0.1681	52	1.47	0.1466	0.9990
**Middle**	**-1**	**Middle**	**2**	0.2653	0.1681	52	1.58	0.1204	0.9973
**Middle**	**-1**	**Middle**	**3**	0.2173	0.1681	52	1.29	0.2016	0.9999
**Middle**	**-1**	**Middle**	**4**	0.2820	0.1681	52	1.68	0.0993	0.9937
**Middle**	**-1**	**Middle**	**5**	0.2840	0.1681	52	1.69	0.0970	0.9931
**Middle**	**-1**	**Middle**	**6**	0.3900	0.1681	52	2.32	0.0243	0.8291
**Middle**	**-1**	**Middle**	**7**	0.4960	0.1681	52	2.95	0.0047	0.4066
**Middle**	**-1**	**Out**	**-1**	-0.02800	0.1681	52	-0.17	0.8683	1.0000
**Middle**	**-1**	**Out**	**0**	0.4620	0.1681	52	2.75	0.0082	0.5465
**Middle**	**-1**	**Out**	**1**	0.4867	0.1681	52	2.90	0.0055	0.4437
**Middle**	**-1**	**Out**	**2**	0.5137	0.1681	52	3.06	0.0035	0.3406
**Middle**	**-1**	**Out**	**3**	0.5827	0.1681	52	3.47	0.0011	0.1484
**Middle**	**-1**	**Out**	**4**	0.5870	0.1681	52	3.49	0.0010	0.1399
**Middle**	**-1**	**Out**	**5**	0.5907	0.1681	52	3.51	0.0009	0.1330
**Middle**	**-1**	**Out**	**6**	0.8410	0.1681	52	5.00	<.0001	0.0018
**Middle**	**-1**	**Out**	**7**	1.0580	0.1681	52	6.30	<.0001	<.0001
**Middle**	**0**	**Middle**	**1**	0.1357	0.1681	52	0.81	0.4232	1.0000
**Middle**	**0**	**Middle**	**2**	0.1533	0.1681	52	0.91	0.3658	1.0000
**Middle**	**0**	**Middle**	**3**	0.1053	0.1681	52	0.63	0.5335	1.0000
**Middle**	**0**	**Middle**	**4**	0.1700	0.1681	52	1.01	0.3164	1.0000
**Middle**	**0**	**Middle**	**5**	0.1720	0.1681	52	1.02	0.3108	1.0000
**Middle**	**0**	**Middle**	**6**	0.2780	0.1681	52	1.65	0.1041	0.9948
**Middle**	**0**	**Middle**	**7**	0.3840	0.1681	52	2.29	0.0264	0.8476
**Middle**	**0**	**Out**	**-1**	-0.1400	0.1681	52	-0.83	0.4086	1.0000
**Middle**	**0**	**Out**	**0**	0.3500	0.1681	52	2.08	0.0422	0.9299

**Middle**	**0**	**Out**	**1**	0.3747	0.1681	52	2.23	0.0301	0.8740
**Middle**	**0**	**Out**	**2**	0.4017	0.1681	52	2.39	0.0205	0.7900
**Middle**	**0**	**Out**	**3**	0.4707	0.1681	52	2.80	0.0071	0.5098
**Middle**	**0**	**Out**	**4**	0.4750	0.1681	52	2.83	0.0067	0.4917
**Middle**	**0**	**Out**	**5**	0.4787	0.1681	52	2.85	0.0063	0.4764
**Middle**	**0**	**Out**	**6**	0.7290	0.1681	52	4.34	<.0001	0.0146
**Middle**	**0**	**Out**	**7**	0.9460	0.1681	52	5.63	<.0001	0.0002
**Middle**	**1**	**Middle**	**2**	0.01767	0.1681	52	0.11	0.9167	1.0000
**Middle**	**1**	**Middle**	**3**	-0.03033	0.1681	52	-0.18	0.8575	1.0000
**Middle**	**1**	**Middle**	**4**	0.03433	0.1681	52	0.20	0.8389	1.0000
**Middle**	**1**	**Middle**	**5**	0.03633	0.1681	52	0.22	0.8297	1.0000
**Middle**	**1**	**Middle**	**6**	0.1423	0.1681	52	0.85	0.4009	1.0000
**Middle**	**1**	**Middle**	**7**	0.2483	0.1681	52	1.48	0.1455	0.9990
**Middle**	**1**	**Out**	**-1**	-0.2757	0.1681	52	-1.64	0.1070	0.9954
**Middle**	**1**	**Out**	**0**	0.2143	0.1681	52	1.28	0.2078	0.9999
**Middle**	**1**	**Out**	**1**	0.2390	0.1681	52	1.42	0.1609	0.9994
**Middle**	**1**	**Out**	**2**	0.2660	0.1681	52	1.58	0.1195	0.9972
**Middle**	**1**	**Out**	**3**	0.3350	0.1681	52	1.99	0.0515	0.9540
**Middle**	**1**	**Out**	**4**	0.3393	0.1681	52	2.02	0.0486	0.9478
**Middle**	**1**	**Out**	**5**	0.3430	0.1681	52	2.04	0.0463	0.9420
**Middle**	**1**	**Out**	**6**	0.5933	0.1681	52	3.53	0.0009	0.1282
**Middle**	**1**	**Out**	**7**	0.8103	0.1681	52	4.82	<.0001	0.0032
**Middle**	**2**	**Middle**	**3**	-0.04800	0.1681	52	-0.29	0.7763	1.0000
**Middle**	**2**	**Middle**	**4**	0.01667	0.1681	52	0.10	0.9214	1.0000
**Middle**	**2**	**Middle**	**5**	0.01867	0.1681	52	0.11	0.9120	1.0000
**Middle**	**2**	**Middle**	**6**	0.1247	0.1681	52	0.74	0.4615	1.0000
**Middle**	**2**	**Middle**	**7**	0.2307	0.1681	52	1.37	0.1758	0.9997
**Middle**	**2**	**Out**	**-1**	-0.2933	0.1681	52	-1.75	0.0868	0.9896
**Middle**	**2**	**Out**	**0**	0.1967	0.1681	52	1.17	0.2472	1.0000
**Middle**	**2**	**Out**	**1**	0.2213	0.1681	52	1.32	0.1936	0.9998
**Middle**	**2**	**Out**	**2**	0.2483	0.1681	52	1.48	0.1455	0.9990
**Middle**	**2**	**Out**	**3**	0.3173	0.1681	52	1.89	0.0646	0.9740
**Middle**	**2**	**Out**	**4**	0.3217	0.1681	52	1.91	0.0611	0.9698

**Middle**	**2**	**Out**	**5**	0.3253	0.1681	52	1.94	0.0583	0.9660
**Middle**	**2**	**Out**	**6**	0.5757	0.1681	52	3.43	0.0012	0.1629
**Middle**	**2**	**Out**	**7**	0.7927	0.1681	52	4.72	<.0001	0.0045
**Middle**	**3**	**Middle**	**4**	0.06467	0.1681	52	0.38	0.7020	1.0000
**Middle**	**3**	**Middle**	**5**	0.06667	0.1681	52	0.40	0.6932	1.0000
**Middle**	**3**	**Middle**	**6**	0.1727	0.1681	52	1.03	0.3090	1.0000
**Middle**	**3**	**Middle**	**7**	0.2787	0.1681	52	1.66	0.1033	0.9946
**Middle**	**3**	**Out**	**-1**	-0.2453	0.1681	52	-1.46	0.1503	0.9991
**Middle**	**3**	**Out**	**0**	0.2447	0.1681	52	1.46	0.1514	0.9992
**Middle**	**3**	**Out**	**1**	0.2693	0.1681	52	1.60	0.1151	0.9966
**Middle**	**3**	**Out**	**2**	0.2963	0.1681	52	1.76	0.0837	0.9883
**Middle**	**3**	**Out**	**3**	0.3653	0.1681	52	2.17	0.0343	0.8976
**Middle**	**3**	**Out**	**4**	0.3697	0.1681	52	2.20	0.0323	0.8870
**Middle**	**3**	**Out**	**5**	0.3733	0.1681	52	2.22	0.0307	0.8776
**Middle**	**3**	**Out**	**6**	0.6237	0.1681	52	3.71	0.0005	0.0828
**Middle**	**3**	**Out**	**7**	0.8407	0.1681	52	5.00	<.0001	0.0018
**Middle**	**4**	**Middle**	**5**	0.002000	0.1681	52	0.01	0.9906	1.0000
**Middle**	**4**	**Middle**	**6**	0.1080	0.1681	52	0.64	0.5233	1.0000
**Middle**	**4**	**Middle**	**7**	0.2140	0.1681	52	1.27	0.2085	0.9999
**Middle**	**4**	**Out**	**-1**	-0.3100	0.1681	52	-1.84	0.0708	0.9800
**Middle**	**4**	**Out**	**0**	0.1800	0.1681	52	1.07	0.2891	1.0000
**Middle**	**4**	**Out**	**1**	0.2047	0.1681	52	1.22	0.2288	1.0000
**Middle**	**4**	**Out**	**2**	0.2317	0.1681	52	1.38	0.1739	0.9996
**Middle**	**4**	**Out**	**3**	0.3007	0.1681	52	1.79	0.0794	0.9860
**Middle**	**4**	**Out**	**4**	0.3050	0.1681	52	1.81	0.0753	0.9834
**Middle**	**4**	**Out**	**5**	0.3087	0.1681	52	1.84	0.0720	0.9810
**Middle**	**4**	**Out**	**6**	0.5590	0.1681	52	3.33	0.0016	0.2019
**Middle**	**4**	**Out**	**7**	0.7760	0.1681	52	4.62	<.0001	0.0062
**Middle**	**5**	**Middle**	**6**	0.1060	0.1681	52	0.63	0.5310	1.0000
**Middle**	**5**	**Middle**	**7**	0.2120	0.1681	52	1.26	0.2128	0.9999
**Middle**	**5**	**Out**	**-1**	-0.3120	0.1681	52	-1.86	0.0690	0.9785
**Middle**	**5**	**Out**	**0**	0.1780	0.1681	52	1.06	0.2944	1.0000
**Middle**	**5**	**Out**	**1**	0.2027	0.1681	52	1.21	0.2333	1.0000

**Middle**	**5**	**Out**	**2**	0.2297	0.1681	52	1.37	0.1776	0.9997
**Middle**	**5**	**Out**	**3**	0.2987	0.1681	52	1.78	0.0814	0.9871
**Middle**	**5**	**Out**	**4**	0.3030	0.1681	52	1.80	0.0772	0.9847
**Middle**	**5**	**Out**	**5**	0.3067	0.1681	52	1.82	0.0738	0.9823
**Middle**	**5**	**Out**	**6**	0.5570	0.1681	52	3.31	0.0017	0.2070
**Middle**	**5**	**Out**	**7**	0.7740	0.1681	52	4.61	<.0001	0.0064
**Middle**	**6**	**Middle**	**7**	0.1060	0.1681	52	0.63	0.5310	1.0000
**Middle**	**6**	**Out**	**-1**	-0.4180	0.1681	52	-2.49	0.0161	0.7294
**Middle**	**6**	**Out**	**0**	0.07200	0.1681	52	0.43	0.6701	1.0000
**Middle**	**6**	**Out**	**1**	0.09667	0.1681	52	0.58	0.5676	1.0000
**Middle**	**6**	**Out**	**2**	0.1237	0.1681	52	0.74	0.4651	1.0000
**Middle**	**6**	**Out**	**3**	0.1927	0.1681	52	1.15	0.2568	1.0000
**Middle**	**6**	**Out**	**4**	0.1970	0.1681	52	1.17	0.2464	1.0000
**Middle**	**6**	**Out**	**5**	0.2007	0.1681	52	1.19	0.2379	1.0000
**Middle**	**6**	**Out**	**6**	0.4510	0.1681	52	2.68	0.0097	0.5934
**Middle**	**6**	**Out**	**7**	0.6680	0.1681	52	3.97	0.0002	0.0414
**Middle**	**7**	**Out**	**-1**	-0.5240	0.1681	52	-3.12	0.0030	0.3049
**Middle**	**7**	**Out**	**0**	-0.03400	0.1681	52	-0.20	0.8405	1.0000
**Middle**	**7**	**Out**	**1**	-0.00933	0.1681	52	-0.06	0.9559	1.0000
**Middle**	**7**	**Out**	**2**	0.01767	0.1681	52	0.11	0.9167	1.0000
**Middle**	**7**	**Out**	**3**	0.08667	0.1681	52	0.52	0.6082	1.0000
**Middle**	**7**	**Out**	**4**	0.09100	0.1681	52	0.54	0.5905	1.0000
**Middle**	**7**	**Out**	**5**	0.09467	0.1681	52	0.56	0.5756	1.0000
**Middle**	**7**	**Out**	**6**	0.3450	0.1681	52	2.05	0.0451	0.9387
**Middle**	**7**	**Out**	**7**	0.5620	0.1681	52	3.34	0.0015	0.1944
**Out**	**-1**	**Out**	**0**	0.4900	0.1681	52	2.92	0.0052	0.4303
**Out**	**-1**	**Out**	**1**	0.5147	0.1681	52	3.06	0.0035	0.3370
**Out**	**-1**	**Out**	**2**	0.5417	0.1681	52	3.22	0.0022	0.2493
**Out**	**-1**	**Out**	**3**	0.6107	0.1681	52	3.63	0.0006	0.1002
**Out**	**-1**	**Out**	**4**	0.6150	0.1681	52	3.66	0.0006	0.0941
**Out**	**-1**	**Out**	**5**	0.6187	0.1681	52	3.68	0.0006	0.0892
**Out**	**-1**	**Out**	**6**	0.8690	0.1681	52	5.17	<.0001	0.0010
**Out**	**-1**	**Out**	**7**	1.0860	0.1681	52	6.46	<.0001	<.0001

**Out**	**0**	**Out**	**1**	0.02467	0.1681	52	0.15	0.8839	1.0000
**Out**	**0**	**Out**	**2**	0.05167	0.1681	52	0.31	0.7597	1.0000
**Out**	**0**	**Out**	**3**	0.1207	0.1681	52	0.72	0.4759	1.0000
**Out**	**0**	**Out**	**4**	0.1250	0.1681	52	0.74	0.4603	1.0000
**Out**	**0**	**Out**	**5**	0.1287	0.1681	52	0.77	0.4474	1.0000
**Out**	**0**	**Out**	**6**	0.3790	0.1681	52	2.26	0.0284	0.8621
**Out**	**0**	**Out**	**7**	0.5960	0.1681	52	3.55	0.0008	0.1235
**Out**	**1**	**Out**	**2**	0.02700	0.1681	52	0.16	0.8730	1.0000
**Out**	**1**	**Out**	**3**	0.09600	0.1681	52	0.57	0.5703	1.0000
**Out**	**1**	**Out**	**4**	0.1003	0.1681	52	0.60	0.5531	1.0000
**Out**	**1**	**Out**	**5**	0.1040	0.1681	52	0.62	0.5387	1.0000
**Out**	**1**	**Out**	**6**	0.3543	0.1681	52	2.11	0.0398	0.9216
**Out**	**1**	**Out**	**7**	0.5713	0.1681	52	3.40	0.0013	0.1725
**Out**	**2**	**Out**	**3**	0.06900	0.1681	52	0.41	0.6831	1.0000
**Out**	**2**	**Out**	**4**	0.07333	0.1681	52	0.44	0.6644	1.0000
**Out**	**2**	**Out**	**5**	0.07700	0.1681	52	0.46	0.6487	1.0000
**Out**	**2**	**Out**	**6**	0.3273	0.1681	52	1.95	0.0568	0.9637
**Out**	**2**	**Out**	**7**	0.5443	0.1681	52	3.24	0.0021	0.2416
**Out**	**3**	**Out**	**4**	0.004333	0.1681	52	0.03	0.9795	1.0000
**Out**	**3**	**Out**	**5**	0.008000	0.1681	52	0.05	0.9622	1.0000
**Out**	**3**	**Out**	**6**	0.2583	0.1681	52	1.54	0.1303	0.9981
**Out**	**3**	**Out**	**7**	0.4753	0.1681	52	2.83	0.0066	0.4903
**Out**	**4**	**Out**	**5**	0.003667	0.1681	52	0.02	0.9827	1.0000
**Out**	**4**	**Out**	**6**	0.2540	0.1681	52	1.51	0.1367	0.9985
**Out**	**4**	**Out**	**7**	0.4710	0.1681	52	2.80	0.0071	0.5084
**Out**	**5**	**Out**	**6**	0.2503	0.1681	52	1.49	0.1424	0.9988
**Out**	**5**	**Out**	**7**	0.4673	0.1681	52	2.78	0.0075	0.5239
**Out**	**6**	**Out**	**7**	0.2170	0.1681	52	1.29	0.2023	0.9999

**Tukey Grouping for Position*Day Least Squares Means (Alpha=0.05)**			
**LS-means with the same letter are not significantly different.**			
**Position**	**Day**	**Estimate**	

**Heart**	**-1**	2.3493			A	
					A	
**Out**	**-1**	2.2680	B		A	
			B		A	
**Middle**	**-1**	2.2400	B		A	
			B		A	
**Heart**	**1**	2.2377	B		A	
			B		A	
**Heart**	**0**	2.1870	B		A	
			B		A	
**Middle**	**0**	2.1280	B		A	
			B		A	
**Heart**	**6**	2.0993	B		A	
			B		A	
**Heart**	**3**	2.0800	B		A	
			B		A	
**Heart**	**4**	2.0280	B		A	C
			B		A	C
**Middle**	**3**	2.0227	B		A	C
			B		A	C
**Heart**	**7**	1.9977	B		A	C
			B		A	C
**Middle**	**1**	1.9923	B		A	C
			B		A	C
**Heart**	**2**	1.9797	B		A	C
			B		A	C
**Middle**	**2**	1.9747	B		A	C
			B		A	C
**Heart**	**5**	1.9720	B		A	C
			B		A	C
**Middle**	**4**	1.9580	B		A	C
			B		A	C
**Middle**	**5**	1.9560	B		A	C

			B		A	C
**Middle**	**6**	1.8500	B		A	C
			B		A	C
**Out**	**0**	1.7780	B	D	A	C
			B	D	A	C
**Out**	**1**	1.7533	B	D	A	C
			B	D	A	C
**Middle**	**7**	1.7440	B	D	A	C
			B	D	A	C
**Out**	**2**	1.7263	B	D	A	C
			B	D		C
**Out**	**3**	1.6573	B	D		C
			B	D		C
**Out**	**4**	1.6530	B	D		C
			B	D		C
**Out**	**5**	1.6493	B	D		C
				D		C
**Out**	**6**	1.3990		D		C
				D		
**Out**	**7**	1.1820		D		

Strawberry Data

The SAS System

The GLIMMIX Procedure

**Model Information**	
**Data Set**	WORK.PWSSTRAWBERRY
**Response Variable**	log
**Response Distribution**	Gaussian
**Link Function**	Identity
**Variance Function**	Default
**Variance Matrix**	Not blocked

**Estimation Technique**	Restricted Maximum Likelihood
**Degrees of Freedom Method**	Containment

Class Level Information		
**Class**	**Levels**	**Values**
**Position**	2	Fruit Leaf
**Day**	8	-1 0 1 2 3 4 5 6
**Rep**	3	1 2 3

**Number of Observations Read**	48
**Number of Observations Used**	48

Dimensions	
**G-side Cov. Parameters**	2
**R-side Cov. Parameters**	1
**Columns in X**	30
**Columns in Z**	3
**Subjects (Blocks in V)**	1
**Max Obs per Subject**	48

Optimization Information	
**Optimization Technique**	Dual Quasi-Newton
**Parameters in Optimization**	2
**Lower Boundaries**	1
**Upper Boundaries**	0
**Fixed Effects**	Profiled
**Residual Variance**	Profiled
**Starting From**	Data

Iteration History					
**Iteration**	**Restarts**	**Evaluations**	**Objective Function**	**Change**	**Max Gradient**
**0**	**0**	4	75.228037583	.	2.84E-14

Convergence criterion (ABSGCONV=0.00001) satisfied.

Estimated G matrix is not positive definite.

Fit Statistics	
**-2 Res Log Likelihood**	75.23
**AIC (smaller is better)**	79.23
**AICC (smaller is better)**	79.67
**BIC (smaller is better)**	77.43
**CAIC (smaller is better)**	79.43
**HQIC (smaller is better)**	75.60
**Generalized Chi-Square**	10.35
**Gener. Chi-Square / DF**	0.34

Covariance Parameter Estimates		
**Cov Parm**	**Estimate**	**Standard Error**
**Variance**	0	.
**CS**	0	511496
**Residual**	0.3449	0.08906

Type III Tests of Fixed Effects				
**Effect**	**Num DF**	**Den DF**	**F Value**	**Pr > F**
**Position**	1	30	60.66	<.0001
**Day**	7	30	9.64	<.0001
**Position*Day**	7	30	1.47	0.2157
**Rep**	2	0	0.06	.

Position Least Squares Means					
**Position**	**Estimate**	**Standard Error**	**DF**	**t Value**	**Pr > |t|**
**Fruit**	0.2988	0.1199	30	2.49	0.0185
**Leaf**	1.6193	0.1199	30	13.51	<.0001

Differences of Position Least Squares Means<br>Adjustment for Multiple Comparisons: Tukey							
**Position**	**_Position**	**Estimate**	**Standard Error**	**DF**	**t Value**	**Pr > |t|**	**Adj P**
**Fruit**	**Leaf**	-1.3205	0.1695	30	-7.79	<.0001	<.0001

Tukey Grouping for Position Least Squares Means (Alpha=0.05)		
LS-means with the same letter are not significantly different.		
**Position**	**Estimate**	
**Leaf**	1.6193	A
**Fruit**	0.2988	B

Day Least Squares Means					
**Day**	**Estimate**	**Standard Error**	**DF**	**t Value**	**Pr > |t|**
**-1**	2.0562	0.2398	30	8.58	<.0001
**0**	1.8320	0.2398	30	7.64	<.0001
**1**	1.3667	0.2398	30	5.70	<.0001
**2**	0.7968	0.2398	30	3.32	0.0024
**3**	0.9305	0.2398	30	3.88	0.0005
**4**	0.5135	0.2398	30	2.14	0.0405
**5**	0.1345	0.2398	30	0.56	0.5790
**6**	0.04183	0.2398	30	0.17	0.8627

Differences of Day Least Squares Means<br>Adjustment for Multiple Comparisons: Tukey							
**Day**	**_Day**	**Estimate**	**Standard Error**	**DF**	**t Value**	**Pr > |t|**	**Adj P**
**-1**	**0**	0.2242	0.3391	30	0.66	0.5136	0.9975
**-1**	**1**	0.6895	0.3391	30	2.03	0.0509	0.4778
**-1**	**2**	1.2593	0.3391	30	3.71	0.0008	0.0166
**-1**	**3**	1.1257	0.3391	30	3.32	0.0024	0.0429
**-1**	**4**	1.5427	0.3391	30	4.55	<.0001	0.0019

**-1**	**5**	1.9217	0.3391	30	5.67	<.0001	<.0001
**-1**	**6**	2.0143	0.3391	30	5.94	<.0001	<.0001
**0**	**1**	0.4653	0.3391	30	1.37	0.1801	0.8624
**0**	**2**	1.0352	0.3391	30	3.05	0.0047	0.0783
**0**	**3**	0.9015	0.3391	30	2.66	0.0125	0.1750
**0**	**4**	1.3185	0.3391	30	3.89	0.0005	0.0107
**0**	**5**	1.6975	0.3391	30	5.01	<.0001	0.0005
**0**	**6**	1.7902	0.3391	30	5.28	<.0001	0.0003
**1**	**2**	0.5698	0.3391	30	1.68	0.1032	0.6990
**1**	**3**	0.4362	0.3391	30	1.29	0.2082	0.8968
**1**	**4**	0.8532	0.3391	30	2.52	0.0174	0.2273
**1**	**5**	1.2322	0.3391	30	3.63	0.0010	0.0202
**1**	**6**	1.3248	0.3391	30	3.91	0.0005	0.0102
**2**	**3**	-0.1337	0.3391	30	-0.39	0.6962	0.9999
**2**	**4**	0.2833	0.3391	30	0.84	0.4100	0.9894
**2**	**5**	0.6623	0.3391	30	1.95	0.0602	0.5276
**2**	**6**	0.7550	0.3391	30	2.23	0.0336	0.3652
**3**	**4**	0.4170	0.3391	30	1.23	0.2283	0.9164
**3**	**5**	0.7960	0.3391	30	2.35	0.0257	0.3025
**3**	**6**	0.8887	0.3391	30	2.62	0.0136	0.1879
**4**	**5**	0.3790	0.3391	30	1.12	0.2726	0.9477
**4**	**6**	0.4717	0.3391	30	1.39	0.1745	0.8542
**5**	**6**	0.09267	0.3391	30	0.27	0.7865	1.0000

**Tukey Grouping for Day Least Squares Means (Alpha=0.05)**				
**LS-means with the same letter are not significantly different.**				
**Day**	**Estimate**			
**-1**	2.0562		A	
			A	
**0**	1.8320	B	A	
		B	A	

**1**	1.3667	B	A	C
		B		C
**3**	0.9305	B	D	C
		B	D	C
**2**	0.7968	B	D	C
			D	C
**4**	0.5135		D	C
			D	
**5**	0.1345		D	
			D	
**6**	0.04183		D	

Position*Day Least Squares Means						
**Position**	**Day**	**Estimate**	**Standard Error**	**DF**	**t Value**	**Pr > |t|**
**Fruit**	**-1**	1.6047	0.3391	30	4.73	<.0001
**Fruit**	**0**	1.5830	0.3391	30	4.67	<.0001
**Fruit**	**1**	0.7743	0.3391	30	2.28	0.0296
**Fruit**	**2**	-0.02567	0.3391	30	-0.08	0.9402
**Fruit**	**3**	0.5240	0.3391	30	1.55	0.1327
**Fruit**	**4**	-0.4873	0.3391	30	-1.44	0.1610
**Fruit**	**5**	-0.5567	0.3391	30	-1.64	0.1111
**Fruit**	**6**	-1.0263	0.3391	30	-3.03	0.0050
**Leaf**	**-1**	2.5077	0.3391	30	7.40	<.0001
**Leaf**	**0**	2.0810	0.3391	30	6.14	<.0001
**Leaf**	**1**	1.9590	0.3391	30	5.78	<.0001
**Leaf**	**2**	1.6193	0.3391	30	4.78	<.0001
**Leaf**	**3**	1.3370	0.3391	30	3.94	0.0004
**Leaf**	**4**	1.5143	0.3391	30	4.47	0.0001
**Leaf**	**5**	0.8257	0.3391	30	2.44	0.0211
**Leaf**	**6**	1.1100	0.3391	30	3.27	0.0027

Differences of Position*Day Least Squares Means<br>Adjustment for Multiple Comparisons: Tukey

Position	Day	_Position	_Day	Estimate	Standard Error	DF	t Value	Pr > |t|	Adj P
**Fruit**	**-1**	**Fruit**	**0**	0.02167	0.4795	30	0.05	0.9643	1.0000
**Fruit**	**-1**	**Fruit**	**1**	0.8303	0.4795	30	1.73	0.0936	0.9214
**Fruit**	**-1**	**Fruit**	**2**	1.6303	0.4795	30	3.40	0.0019	0.1034
**Fruit**	**-1**	**Fruit**	**3**	1.0807	0.4795	30	2.25	0.0317	0.6565
**Fruit**	**-1**	**Fruit**	**4**	2.0920	0.4795	30	4.36	0.0001	0.0106
**Fruit**	**-1**	**Fruit**	**5**	2.1613	0.4795	30	4.51	<.0001	0.0073
**Fruit**	**-1**	**Fruit**	**6**	2.6310	0.4795	30	5.49	<.0001	0.0005
**Fruit**	**-1**	**Leaf**	**-1**	-0.9030	0.4795	30	-1.88	0.0694	0.8639
**Fruit**	**-1**	**Leaf**	**0**	-0.4763	0.4795	30	-0.99	0.3285	0.9996
**Fruit**	**-1**	**Leaf**	**1**	-0.3543	0.4795	30	-0.74	0.4657	1.0000
**Fruit**	**-1**	**Leaf**	**2**	-0.01467	0.4795	30	-0.03	0.9758	1.0000
**Fruit**	**-1**	**Leaf**	**3**	0.2677	0.4795	30	0.56	0.5809	1.0000
**Fruit**	**-1**	**Leaf**	**4**	0.09033	0.4795	30	0.19	0.8518	1.0000
**Fruit**	**-1**	**Leaf**	**5**	0.7790	0.4795	30	1.62	0.1147	0.9506
**Fruit**	**-1**	**Leaf**	**6**	0.4947	0.4795	30	1.03	0.3105	0.9994
**Fruit**	**0**	**Fruit**	**1**	0.8087	0.4795	30	1.69	0.1021	0.9348
**Fruit**	**0**	**Fruit**	**2**	1.6087	0.4795	30	3.35	0.0022	0.1137
**Fruit**	**0**	**Fruit**	**3**	1.0590	0.4795	30	2.21	0.0350	0.6853
**Fruit**	**0**	**Fruit**	**4**	2.0703	0.4795	30	4.32	0.0002	0.0119
**Fruit**	**0**	**Fruit**	**5**	2.1397	0.4795	30	4.46	0.0001	0.0082
**Fruit**	**0**	**Fruit**	**6**	2.6093	0.4795	30	5.44	<.0001	0.0006
**Fruit**	**0**	**Leaf**	**-1**	-0.9247	0.4795	30	-1.93	0.0633	0.8432
**Fruit**	**0**	**Leaf**	**0**	-0.4980	0.4795	30	-1.04	0.3073	0.9993
**Fruit**	**0**	**Leaf**	**1**	-0.3760	0.4795	30	-0.78	0.4391	1.0000
**Fruit**	**0**	**Leaf**	**2**	-0.03633	0.4795	30	-0.08	0.9401	1.0000
**Fruit**	**0**	**Leaf**	**3**	0.2460	0.4795	30	0.51	0.6117	1.0000
**Fruit**	**0**	**Leaf**	**4**	0.06867	0.4795	30	0.14	0.8871	1.0000
**Fruit**	**0**	**Leaf**	**5**	0.7573	0.4795	30	1.58	0.1248	0.9603
**Fruit**	**0**	**Leaf**	**6**	0.4730	0.4795	30	0.99	0.3318	0.9996
**Fruit**	**1**	**Fruit**	**2**	0.8000	0.4795	30	1.67	0.1057	0.9397
**Fruit**	**1**	**Fruit**	**3**	0.2503	0.4795	30	0.52	0.6055	1.0000
**Fruit**	**1**	**Fruit**	**4**	1.2617	0.4795	30	2.63	0.0133	0.4153

**Fruit**	**1**	**Fruit**	**5**	1.3310	0.4795	30	2.78	0.0094	0.3335
**Fruit**	**1**	**Fruit**	**6**	1.8007	0.4795	30	3.76	0.0007	0.0469
**Fruit**	**1**	**Leaf**	**-1**	-1.7333	0.4795	30	-3.61	0.0011	0.0646
**Fruit**	**1**	**Leaf**	**0**	-1.3067	0.4795	30	-2.72	0.0106	0.3611
**Fruit**	**1**	**Leaf**	**1**	-1.1847	0.4795	30	-2.47	0.0194	0.5154
**Fruit**	**1**	**Leaf**	**2**	-0.8450	0.4795	30	-1.76	0.0882	0.9113
**Fruit**	**1**	**Leaf**	**3**	-0.5627	0.4795	30	-1.17	0.2499	0.9974
**Fruit**	**1**	**Leaf**	**4**	-0.7400	0.4795	30	-1.54	0.1333	0.9670
**Fruit**	**1**	**Leaf**	**5**	-0.05133	0.4795	30	-0.11	0.9155	1.0000
**Fruit**	**1**	**Leaf**	**6**	-0.3357	0.4795	30	-0.70	0.4893	1.0000
**Fruit**	**2**	**Fruit**	**3**	-0.5497	0.4795	30	-1.15	0.2608	0.9980
**Fruit**	**2**	**Fruit**	**4**	0.4617	0.4795	30	0.96	0.3434	0.9997
**Fruit**	**2**	**Fruit**	**5**	0.5310	0.4795	30	1.11	0.2770	0.9986
**Fruit**	**2**	**Fruit**	**6**	1.0007	0.4795	30	2.09	0.0455	0.7591
**Fruit**	**2**	**Leaf**	**-1**	-2.5333	0.4795	30	-5.28	<.0001	0.0009
**Fruit**	**2**	**Leaf**	**0**	-2.1067	0.4795	30	-4.39	0.0001	0.0098
**Fruit**	**2**	**Leaf**	**1**	-1.9847	0.4795	30	-4.14	0.0003	0.0186
**Fruit**	**2**	**Leaf**	**2**	-1.6450	0.4795	30	-3.43	0.0018	0.0969
**Fruit**	**2**	**Leaf**	**3**	-1.3627	0.4795	30	-2.84	0.0080	0.2995
**Fruit**	**2**	**Leaf**	**4**	-1.5400	0.4795	30	-3.21	0.0031	0.1522
**Fruit**	**2**	**Leaf**	**5**	-0.8513	0.4795	30	-1.78	0.0860	0.9067
**Fruit**	**2**	**Leaf**	**6**	-1.1357	0.4795	30	-2.37	0.0245	0.5819
**Fruit**	**3**	**Fruit**	**4**	1.0113	0.4795	30	2.11	0.0434	0.7461
**Fruit**	**3**	**Fruit**	**5**	1.0807	0.4795	30	2.25	0.0317	0.6565
**Fruit**	**3**	**Fruit**	**6**	1.5503	0.4795	30	3.23	0.0030	0.1458
**Fruit**	**3**	**Leaf**	**-1**	-1.9837	0.4795	30	-4.14	0.0003	0.0187
**Fruit**	**3**	**Leaf**	**0**	-1.5570	0.4795	30	-3.25	0.0029	0.1418
**Fruit**	**3**	**Leaf**	**1**	-1.4350	0.4795	30	-2.99	0.0055	0.2306
**Fruit**	**3**	**Leaf**	**2**	-1.0953	0.4795	30	-2.28	0.0296	0.6368
**Fruit**	**3**	**Leaf**	**3**	-0.8130	0.4795	30	-1.70	0.1004	0.9323
**Fruit**	**3**	**Leaf**	**4**	-0.9903	0.4795	30	-2.07	0.0476	0.7715
**Fruit**	**3**	**Leaf**	**5**	-0.3017	0.4795	30	-0.63	0.5341	1.0000
**Fruit**	**3**	**Leaf**	**6**	-0.5860	0.4795	30	-1.22	0.2312	0.9961

**Fruit**	**4**	**Fruit**	**5**	0.06933	0.4795	30	0.14	0.8860	1.0000
**Fruit**	**4**	**Fruit**	**6**	0.5390	0.4795	30	1.12	0.2699	0.9984
**Fruit**	**4**	**Leaf**	**-1**	-2.9950	0.4795	30	-6.25	<.0001	<.0001
**Fruit**	**4**	**Leaf**	**0**	-2.5683	0.4795	30	-5.36	<.0001	0.0008
**Fruit**	**4**	**Leaf**	**1**	-2.4463	0.4795	30	-5.10	<.0001	0.0015
**Fruit**	**4**	**Leaf**	**2**	-2.1067	0.4795	30	-4.39	0.0001	0.0098
**Fruit**	**4**	**Leaf**	**3**	-1.8243	0.4795	30	-3.80	0.0007	0.0418
**Fruit**	**4**	**Leaf**	**4**	-2.0017	0.4795	30	-4.17	0.0002	0.0171
**Fruit**	**4**	**Leaf**	**5**	-1.3130	0.4795	30	-2.74	0.0103	0.3538
**Fruit**	**4**	**Leaf**	**6**	-1.5973	0.4795	30	-3.33	0.0023	0.1194
**Fruit**	**5**	**Fruit**	**6**	0.4697	0.4795	30	0.98	0.3352	0.9996
**Fruit**	**5**	**Leaf**	**-1**	-3.0643	0.4795	30	-6.39	<.0001	<.0001
**Fruit**	**5**	**Leaf**	**0**	-2.6377	0.4795	30	-5.50	<.0001	0.0005
**Fruit**	**5**	**Leaf**	**1**	-2.5157	0.4795	30	-5.25	<.0001	0.0010
**Fruit**	**5**	**Leaf**	**2**	-2.1760	0.4795	30	-4.54	<.0001	0.0068
**Fruit**	**5**	**Leaf**	**3**	-1.8937	0.4795	30	-3.95	0.0004	0.0296
**Fruit**	**5**	**Leaf**	**4**	-2.0710	0.4795	30	-4.32	0.0002	0.0119
**Fruit**	**5**	**Leaf**	**5**	-1.3823	0.4795	30	-2.88	0.0072	0.2796
**Fruit**	**5**	**Leaf**	**6**	-1.6667	0.4795	30	-3.48	0.0016	0.0879
**Fruit**	**6**	**Leaf**	**-1**	-3.5340	0.4795	30	-7.37	<.0001	<.0001
**Fruit**	**6**	**Leaf**	**0**	-3.1073	0.4795	30	-6.48	<.0001	<.0001
**Fruit**	**6**	**Leaf**	**1**	-2.9853	0.4795	30	-6.23	<.0001	<.0001
**Fruit**	**6**	**Leaf**	**2**	-2.6457	0.4795	30	-5.52	<.0001	0.0005
**Fruit**	**6**	**Leaf**	**3**	-2.3633	0.4795	30	-4.93	<.0001	0.0024
**Fruit**	**6**	**Leaf**	**4**	-2.5407	0.4795	30	-5.30	<.0001	0.0009
**Fruit**	**6**	**Leaf**	**5**	-1.8520	0.4795	30	-3.86	0.0006	0.0365
**Fruit**	**6**	**Leaf**	**6**	-2.1363	0.4795	30	-4.46	0.0001	0.0084
**Leaf**	**-1**	**Leaf**	**0**	0.4267	0.4795	30	0.89	0.3807	0.9999
**Leaf**	**-1**	**Leaf**	**1**	0.5487	0.4795	30	1.14	0.2616	0.9980
**Leaf**	**-1**	**Leaf**	**2**	0.8883	0.4795	30	1.85	0.0738	0.8771
**Leaf**	**-1**	**Leaf**	**3**	1.1707	0.4795	30	2.44	0.0208	0.5343
**Leaf**	**-1**	**Leaf**	**4**	0.9933	0.4795	30	2.07	0.0470	0.7679
**Leaf**	**-1**	**Leaf**	**5**	1.6820	0.4795	30	3.51	0.0014	0.0820

**Leaf**	**-1**	**Leaf**	**6**	1.3977	0.4795	30	2.91	0.0067	0.2647
**Leaf**	**0**	**Leaf**	**1**	0.1220	0.4795	30	0.25	0.8009	1.0000
**Leaf**	**0**	**Leaf**	**2**	0.4617	0.4795	30	0.96	0.3434	0.9997
**Leaf**	**0**	**Leaf**	**3**	0.7440	0.4795	30	1.55	0.1313	0.9655
**Leaf**	**0**	**Leaf**	**4**	0.5667	0.4795	30	1.18	0.2466	0.9972
**Leaf**	**0**	**Leaf**	**5**	1.2553	0.4795	30	2.62	0.0137	0.4232
**Leaf**	**0**	**Leaf**	**6**	0.9710	0.4795	30	2.02	0.0519	0.7939
**Leaf**	**1**	**Leaf**	**2**	0.3397	0.4795	30	0.71	0.4842	1.0000
**Leaf**	**1**	**Leaf**	**3**	0.6220	0.4795	30	1.30	0.2045	0.9929
**Leaf**	**1**	**Leaf**	**4**	0.4447	0.4795	30	0.93	0.3612	0.9998
**Leaf**	**1**	**Leaf**	**5**	1.1333	0.4795	30	2.36	0.0248	0.5851
**Leaf**	**1**	**Leaf**	**6**	0.8490	0.4795	30	1.77	0.0868	0.9084
**Leaf**	**2**	**Leaf**	**3**	0.2823	0.4795	30	0.59	0.5604	1.0000
**Leaf**	**2**	**Leaf**	**4**	0.1050	0.4795	30	0.22	0.8282	1.0000
**Leaf**	**2**	**Leaf**	**5**	0.7937	0.4795	30	1.66	0.1083	0.9432
**Leaf**	**2**	**Leaf**	**6**	0.5093	0.4795	30	1.06	0.2966	0.9991
**Leaf**	**3**	**Leaf**	**4**	-0.1773	0.4795	30	-0.37	0.7141	1.0000
**Leaf**	**3**	**Leaf**	**5**	0.5113	0.4795	30	1.07	0.2948	0.9991
**Leaf**	**3**	**Leaf**	**6**	0.2270	0.4795	30	0.47	0.6394	1.0000
**Leaf**	**4**	**Leaf**	**5**	0.6887	0.4795	30	1.44	0.1613	0.9820
**Leaf**	**4**	**Leaf**	**6**	0.4043	0.4795	30	0.84	0.4058	0.9999
**Leaf**	**5**	**Leaf**	**6**	-0.2843	0.4795	30	-0.59	0.5577	1.0000

**Tukey Grouping for Position*Day Least Squares Means (Alpha=0.05)**						
**LS-means with the same letter are not significantly different.**						
**Position**	**Day**	**Estimate**				
**Leaf**	**-1**	2.5077			A	
					A	
**Leaf**	**0**	2.0810	B		A	
			B		A	
**Leaf**	**1**	1.9590	B		A	
			B		A	

**Leaf**	**2**	1.6193	B		A	C
			B		A	C
**Fruit**	**-1**	1.6047	B		A	C
			B		A	C
**Fruit**	**0**	1.5830	B		A	C
			B		A	C
**Leaf**	**4**	1.5143	B		A	C
			B		A	C
**Leaf**	**3**	1.3370	B		A	C
			B		A	C
**Leaf**	**6**	1.1100	B	D	A	C
			B	D	A	C
**Leaf**	**5**	0.8257	B	D	A	C
			B	D	A	C
**Fruit**	**1**	0.7743	B	D	A	C
			B	D		C
**Fruit**	**3**	0.5240	B	D	E	C
				D	E	C
**Fruit**	**2**	-0.02567		D	E	C
				D	E	
**Fruit**	**4**	-0.4873		D	E	
				D	E	
**Fruit**	**5**	-0.5567		D	E	
					E	
**Fruit**	**6**	-1.0263			E	
